# Awareness about developmental coordination disorder

**DOI:** 10.3389/fpubh.2024.1345257

**Published:** 2024-02-01

**Authors:** Bert Steenbergen, Ludvík Valtr, Carolyn Dunford, Melissa Prunty, Hidde Bekhuis, Taha Yassine Temlali, Femke van Abswoude, Jessica M. Lust, Griet Warlop, Mireille Augustijn, Bouwien C. M. Smits-Engelsman, Peter H. Wilson

**Affiliations:** ^1^Behavioural Science Institute, Radboud University, Nijmegen, Netherlands; ^2^Faculty of Physical Culture, Palacký University, Olomouc, Czechia; ^3^Department of Health Sciences, Brunel University London, Uxbridge, United Kingdom; ^4^Department of Movement and Sports Sciences, Ghent University, Ghent, Belgium; ^5^Department of Health and Rehabilitation Sciences, Faculty of Health Sciences, University of Cape Town, Cape Town, South Africa; ^6^Department of Physical Activity, Sport and Recreation, Faculty Health Sciences, North-West University, Potchefstroom, South Africa; ^7^Healthy Brain and Mind Research Centre, Australian Catholic University, Melbourne, VIC, Australia

**Keywords:** awareness, developmental, coordination, disorder, childhood

## Abstract

The present paper is designed to promote awareness of DCD outside the academic world. With a prevalence of 5–6% it is one of the most common disorders of child development. It is therefore surprising that so little is known about it among professionals in child healthcare and education. Parents have expressed frustration about this lack of awareness, including the general public. The general aim of this paper was to describe those critical aspects of DCD that will promote awareness.

## Introduction

A brief story of a child with *Developmental Coordination Disorder* gives a sense of their lived experience of DCD and that of their family. John had a burning desire to be involved in sport, outdoor pursuits and even just schoolyard games. However, he found this very challenging: his poor motor skills did not allow it; it was like there was a huge gulf between what he wanted to do and his ability to do it. His teacher and parents first noticed this when he was a young child but hoped that with time and experience, he would grow out of his so called, “clumsiness.” However, the persistence of these difficulties and the experience of failure had started to impact his motivation to even try to participate. So much so, he began to withdraw from others and retreated from activity which started to affect his fitness, energy, and confidence. His parents were concerned, but what to do?

John’s parents faced a sobering fact: there was a lack of awareness of motor problems in children among clinicians, teachers and in broader society. And, without awareness, how could they find the right support!? After meeting with their general practitioner, his parents were encouraged to get the opinion of a pediatric physical therapist or occupational therapist. Eventually, a specialist appointment resulted in a diagnosis of DCD. It was noted that John’s difficulties extended to a range of activities at school and elsewhere that involved motor coordination like dressing, participating in P.E., navigating around his classroom, handwriting, and even keeping up with the class when keyboarding. Although relieved that John’s motor problems had a name, his parents felt that it did not fully capture his difficulties, especially his lack of confidence and daily planning problems. They also discovered that teachers were not aware of DCD and the impact it may have on education. However, his parents stood their ground in getting the right support for John, where ultimately he benefited from therapy that helped improve his skills and realize his goals.

Not all children with motor difficulties are fortunate enough to get support, which underlines the issue we have with Awareness of DCD. DCD is not widely recognized and, to compound this issue, *not necessarily a problem children grow out of!* Our paper will unpack the reality of DCD in more detail, evaluating its diagnosis, the scientific evidence about its expression, underlying neurological basis and associated problems, the health costs of DCD (including socioeconomic), and the best treatment options.

## Why awareness?

The present paper is designed to promote awareness of DCD outside the academic world. With a prevalence of 5–6% it is one of the most common disorders of child development (1). It is therefore surprising that so little is known about it among professionals in child healthcare and education (2). Parents have expressed frustration about this lack of awareness, including the general public (3–6). The general aim of this paper was to describe those critical aspects of DCD that will promote awareness.

While regarded as a “hidden group” in society, interest in DCD has grown enormously from a research perspective. We performed a simple topic search in Web of Science for the 22-year period between 1992 and 2014 [as done by (1)], as well as the 9-year period between 2014 and 2023. Articles on DCD had increased from 1,364 to 2,106. This means that we see increases of 377% (‘DCD’ search term), 480% (‘DCD’ and ‘diagnosis’), 525% (‘DCD’ and ‘intervention’), and 394% (‘DCD’ and ‘risk factors’) in research. Clearly, particular interest in diagnosis and intervention has skyrocketed in the last decade. Notwithstanding this, awareness has improved only minimally outside the academic arena; DCD is still largely underrecognized by health care professionals, despite its prevalence (1, 7). This warrants a call for action. The aim of our paper is to improve awareness of DCD across all sectors of the community, and an appreciation of the various challenges that individuals with DCD face during their life.

## What is DCD?

### Diagnosis

Diagnosis of DCD is based on four criteria according to the Diagnostic and Statistical Manual of Mental Disorders, 5^th^ Edition, Text Revision [DSM-5-TR; (8)], where it is now conceptualized as a neurodevelopmental disorder. DCD is defined by a marked impairment in the ability to acquire age-appropriate motor skills (criterion 1) which interferes significantly with activities of daily living (like self-care skills), academic performance and/or participation in leisure, vocational activities, and play at school (criterion 2). These motor deficits cannot be better explained by intellectual disability or visual impairment (criterion 3) and cannot be attributed to neurological condition affecting movement (criterion 4), (8, 9).

Common features of DCD are slow and inefficient performance of fine-and/or gross-motor movements, but its specific expression can vary. Indeed, DCD is a fairly heterogeneous condition. Recently, in a large database study, Lust et al. (10) showed four subtypes, discriminated on the basis of difficulties in fine-motor performance, gross-motor performance, visual motor integration and/or cognitive abilities (10). In half of the children, evidence was found for generalized motor impairments. As well, DCD commonly co-occurs with other developmental disorders such as autism spectrum disorder (ASD) and attention deficit/hyperactivity disorder (ADHD) (8, 9).

A sobering fact is that for about one-half of those children diagnosed with DCD, the disorder persists well into adolescence and adulthood (11, 12). Early recognition is thus of utmost importance to provide the right support (evidence-based best practice intervention methods for DCD) and address the social and attitudinal context. Because DCD is still largely unknown among parents and relevant professionals, such as teachers and general practitioners (6, 7, 13), the pathway to diagnosis and treatment can be long and frustrating (5, 6).

### Only a motor problem?

The motor difficulties that are core to DCD diagnosis often have a more generalized impact on development such that academic achievement, inter-personal relationships, employment, poor physical health and fitness (14), and overall wellbeing are also impacted (15–17). Not surprisingly, restricted participation in leisure activities or organized sports is common in DCD. Studies of children and adolescents with DCD have shown that reduced physical activity is not only the result of internal constraints (such as poor skill), but also external constraints (such as ease of access to facilities) that result from a lack of understanding of DCD (18). The lived experience of motor problems implies not meeting the motor performance “norms” of their peers, which can have a negative impact on self-concept, and may lead to bullying and exclusion (19).

The labored and poorly coordinated movements of children with DCD are not due to a lack of effort, but rather are likely to reflect a neurodevelopmental issue that impacts motor-cognitive systems (20). Although the precise etiology of DCD is still debated, researchers worldwide have been trying to identify neurocognitive mechanisms that may underlie the disorder. In a recent review and meta-analysis, the most profound and fundamental deficits were in visual-motor mapping and cognitive-motor integration, showing the important role of cognitive control (e.g., planning, inhibition, shifting) in motor behavior (21). Moreover, there is converging evidence of difficulties with the predictive control of movements, termed the *internal modeling deficit* (IMD) hypothesis (22). These motor control and planning problems are particularly evident when the complexity of an action task increases. Supporting the link between motor and cognitive control factors, a growing number of empirical studies suggest that motor planning problems can be supported through training based on one or a combination of motor imagery/mental rehearsal plus action observation, or using cognitive-based approaches like CO-OP (23). Individuals with DCD may develop adaptive strategies and coping mechanisms to navigate their challenges (24). These may include finding alternative ways to complete tasks or relying on compensatory strategies. However, these strategies may require ongoing effort and may not fully eliminate the impact of DCD on daily life.

Both cross-sectional and longitudinal data show a strong link between the development of executive function and movement skill in children (25, 26). More recently, Wilson et al. (27), in a large cohort study, showed that the DCD cohort had moderate incidence of executive function deficit (41%) at the first measurement point. More importantly, around 26% of children with persisting DCD (2.5 years later) had executive function deficits relative to age-normative data. In short, children with persisting DCD are at significant risk of executive function issues that interfere with activities of daily living (25–27).

### Neurology

Studies on brain mechanisms have shown evidence of atypical function and structure in children with DCD. The most recent meta-analysis of DCD conducted by Subara-Zukic and colleagues (21) identified a number of intriguing hypotheses about the neural basis of DCD. Adult data is suggestive of network disruptions across parietal–frontal and parieto-cerebellar systems. Functional MRI data on fine-motor and cognitive tasks have also shown reduced activation across these networks in DCD (28), while structural MRI studies using diffusion weighted imaging show reduced connectivity in sensory-motor white matter tracts, including the superior longitudinal fasciculus (29, 30). One hypothesis is that this large fronto-parietal tract that connects anterior and posterior brain centers may be delayed in development in children with DCD (21). Although results need to be interpreted with some caution for methodological reasons (e.g., low sample size, variation in methodology and analysis), the findings are broadly consistent with the hypothesis of a possible parietal-cerebellar disconnection in DCD (20). Intriguingly, this neural network supports predictive motor control, particularly the comparison of forward models with external sensory feedback. Finally, executive function deficits *per se* may be associated with delayed maturation of frontoparietal and frontocerebellar networks in DCD (31). Taken together, combined behavioral and neuroimaging studies suggest disruption to motor-cognitive and perceptual-motor integration in DCD. However, longitudinal modeling is needed to unravel these relationships, and to test causality. In addition to this, it is important to note that research in children and adults need to be evaluated in their own right as the brains of adults and children are qualitatively and quantitatively different.

### The broad consequences of DCD in children

From a young age, people with DCD already experience difficulties in carrying out basic self-maintenance activities (32). In a qualitative investigation involving parents of children with DCD, Summers et al. (33) showed that these children (5–7 years old) mainly struggled to perform simple daily activities such as eating, maintaining personal hygiene and dressing. For instance, the parents reported that they had to personally assist their children in putting on clothes. Parents also reported reasons behind their children’s struggles in getting dressed, which included procrastination, problems with spatial orientation of clothing, object manipulation, inability to use fine-motor skills to button shirts, and a much reduced ability to maintain balance when putting on pants, for example (33). Of all clothing items, children reported that socks were the hardest piece to put on. To avoid the burden of dressing, some older children with DCD (8–9 years old) decided to keep wearing their coats indoors and not take them off. Others decided to wear the outfit the night before school to avoid dressing the next morning. As a consequence, older children with DCD tended to neglect their personal appearance (33). With respect to personal hygiene, Summers and colleagues also showed that most children with DCD relied on their parents’ help to comb their hair, take a shower, dry themselves and to cut their nails (33).

The adverse health outcomes associated with DCD are far reaching, affecting not only people’s physical health but also their emotional (34) and mental well-being (35). Moreover, the challenges that children with DCD face do not end in the home but extend to other environmental contexts such as the playground or classroom where there is typically a total lack of awareness about what DCD is and the challenges it presents. Children and teachers report that performance is hindered when trying to write, use scissors, paint, manipulate objects, kick, catch and throw a ball, or run in a field or playground (14, 36, 37).

The social and emotional consequences of DCD are also palpable. The incidence of social isolation and being the subject of bullying is greater in DCD (35). In general, children can find it more difficult to break into social groups at school, and find universal acceptance among their peers. Importantly, the repeated experience of a mismatch between ‘motor ability and the demands of the task at hand places children with DCD at a greater risk of developing negative quality of life outcomes, including depression (35), anxiety (38), low self-worth (34) and self-isolation (39, 40). These negative psychological, social and environmental health-related quality of life outcomes in youth are known to track into adulthood (11, 12).

### Lifelong challenge

While John was able to avoid the embarrassment of physical education classes as an adolescent, life presented new challenges with age such as learning how to drive. While driving symbolized independence and freedom for many older teenagers, for John, it represented a tangled web of novel movements, spatial awareness, and shifting attention. The thought of maneuvering a vehicle, operating pedals, and navigating through traffic filled him with anxiety. In university classes, John faced another hurdle: his handwriting was barely legible, making it hard to keep up with note-taking, and navigating around a busy campus was taxing. Parties, dancing, and outings became new sources of anxiety and isolation. Fear of judgment and a sense of not fitting in or being “different” took a toll on his self-esteem and confidence. Over the years, John experienced some difficulty securing employment, mainly due to the hindrance caused by his poor manual skills and coordination. Indeed, he found himself ruling out certain career choices if they involved precise physical skills.

DCD typically persists into adulthood, posing lifelong and unique challenges (41) but with the specific impacts varying between people with DCD. During adolescence and adulthood, they may encounter persistent difficulties with academic performance, occupational functioning, and social interactions, all while the underlying motor control deficits persist. As such, movement execution may still appear awkward and slower than that of their peers (11). For example, research has shown adequate walking performance on level terrain in adults with DCD, but some inconsistencies in the overall gait pattern (42). Over more challenging terrains, adults with DCD are disadvantaged even more, resulting in slower and safer walking patterns (43). As well, they use adaptive control strategies with altered gaze patterns, and higher and more proximal visual sampling of the terrain (44).

For manual actions, also, altered control is seen in adults with DCD compared with both typically developing adults and children when making sequential reaching actions (45). A reduced ability to use internal models to predict action consequences was still evident, affecting the fine tuning of these movements in these adults. Also, efficient handwriting remains elusive in young adults with DCD: their handwriting skill is less automated than their age matched peers resulting in the production of fewer words on timed writing tasks (46). Their handwriting is labored and inconsistent (47) resulting in poor scores on composition (48). These difficulties inevitably interfere with their ability to process and write information down in academic or work settings, putting greater demands on their overall functioning in occupational settings.

Altogether, the motor control deficits that underlie DCD are quite persistent in most cases, impacting motor behavior and functioning later in life as well, across various domains (49). Academic tasks that require fine-motor skills and (cognitive) organization, like written expression and note-taking, are particularly challenging. These difficulties can impact educational achievement, job performance and career choices. Socially, adolescents and adults with DCD may face barriers in participating in recreational activities, sports, and group interactions, which can lead to social isolation and difficulty forming and maintaining friendships (50, 51). Additionally, the ongoing motor skill difficulties, coupled with the associated challenges in academic and social domains, can raise the risk of emotional distress, low self-esteem, and anxiety/depression (52, 53). Importantly, adults with DCD are more negatively affected by quality-of-life outcomes associated with DCD than by physical outcomes (54). Therefore, early identification and intervention is important to improve motor control and functioning during childhood, as well as sustained help and support during adolescence and adulthood to prevent secondary physical and mental health problems later in life.

Children with DCD are known to have lower activity levels compared with their peers, resulting in less physically active lifestyles and lower physical fitness levels (18). These children are less likely than their typically developing peers to participate in free-play activities or in organized play, such as team sports (55, 56). Consequently, children with DCD are more likely to select sedentary activities and are less likely to enjoy and participate in physical education classes, leisure, and other activities. This reduced sphere of activity may also limit opportunities to participate in social activities including organized sports, exacerbated by limited knowledge about how to integrate such children into a team environment where they feel safe and accepted (57). It is known that social networks, either offline or online, ranging from peers and parents to teachers and medical professionals can play an important role in bringing about sustainable behavioral change which can, in turn, facilitate participation (58, 59).

A qualitative study by Barnett and colleagues (18) shows the importance of the social network for teenagers with DCD. Interviews with teenagers and their parents showed that despite lower levels of participation in physical activity, most enjoyed a range of physical activities. Apart from individual factors related to motor skill and fatigue, factors related to the social network, such as lack of support from peers and lack of teachers’ knowledge of DCD, and even lack of family engagement in the child’s activities were pointed out as important determinants for their participation. Clearly, the social network surrounding the individual with DCD has a crucial impact on their level of participation.

Up to now, little systematic research has examined how social networks can be used to keep children with DCD physically active and motivated. As an illustration, the results of a meta-analysis by Macdonald-Wallis et al. (60) showed a strong positive relationship between sport participation and physical activity in children and their friends, underlining the importance of the child’s social network. Future research should examine how interventions can use the social network to leverage change in physical activity and participation, leading to an active lifestyle.

### Health issues and health economics

Decreased physical activity is associated with elevated health risks, social isolation, reduced participation in society, and work absences. Although figures differ between countries, the social return of investment for sports and active living for the general population is positive, ranging from 1.89 in the United Kingdom (61), 2.25 in the Netherlands (62) and to 3.56 in Flanders, Belgium (63). This implies that every euro invested by the government in sports and active living results in 1.89-to-3.56-euro societal profit (i.e., lower health care costs, more volunteer work, more social trust, etc.). According to the WHO the global health concern has in 2030 a price tag of 371 billion dollars. This is the impetus to get the public more active, a goal that is even more critical for people with DCD! In the Netherlands, for example, there are estimates regarding the costs of inactivity. Specifically, not complying to the Dutch movement guideline, for someone between 5 to 24 years old leads to an estimated annual cost of between 37,000 and 74,000 euros. Under the assumption that 5% of the population has DCD, inactivity of youngsters with DCD alone will cost Dutch society between 0.55 and 1.1 billion euros. On top of this there are the associated costs of therapy, reduced happiness, smaller social capital and elevated risk of early school dropout.

## How can individuals with DCD be helped?

On the positive side, children and youngsters with DCD can function well. They can find their own strategies for navigating daily life activities and it has been deemed important for practitioners to acknowledge these individual strategies (19). Resilience to the development of secondary problems, like psychological distress and physical inactivity has also been an important topic of recent research. For instance, the *environmental stress hypothesis* describes intrapersonal as well as interpersonal factors that may mediate the relationship between impaired motor skills and internalizing problems (64). Recently, *physical literacy* has been posited as an important determinant in the relation between poor motor skills and physical inactivity (56). Awareness of DCD and its consequences among the significant others of those with DCD is likely to be an important factor in increasing social support (interpersonal) as well as self-worth and (intrapersonal) and physical literacy.

Encouragingly, it has been shown that children with DCD can improve with relatively short interventions delivered by healthcare professionals with the relevant training and expertise (41). Interventions should be based on goals set by the child and family with the aim of improving the child’s activity and participation in motor tasks ranging from everyday living skills to sport and recreation activities. High level evidence has shown that a range of approaches can help, including (but not limited to) task specific training, cognitive orientation to occupational performance (CO-OP), neuromotor task training (NTT) and motor imagery training (41). Where handwriting has been identified as a goal, a combination of task-focused and keyboard training is suggested (41). Also, the use of active video gaming has shown early, promising results and may be used as an adjunct to more traditional approaches (41).

Group interventions, with 4–6 children with a therapist and assistant if needed, have been shown to produce large effects on motor performance but this approach would benefit from further research (41, 65). More research is required to be confident about the optimum scheduling, intensity and dose of intervention required but studies to date that show positive outcomes ranged from 2 to 18 weeks with an average of 10 weeks (41). Longer intervention protocols (20–30 h) did not seem more effective that shorter (10–15 h) ones (41).

## Conclusion

Children with DCD face challenges that transcend the motor domain and impact activities of daily living and occupational functioning over the lifespan. DCD puts them at greater risk of secondary health conditions that span physiological fitness and psychosocial well-being. The lives of children with DCD can be improved significantly by the people around them understanding the condition and providing appropriate support. A prerequisite for this to happen is a widespread level of awareness of DCD, similar to other conditions, such ADHD and ASD. Work to date has paved the way for innovation in intervention for the future. However, we need education and health agencies on side to support this work, both financially and from a practitioner training perspective.

## Data availability statement

The original contributions presented in the study are included in the article/supplementary material, further inquiries can be directed to the corresponding author.

## Author contributions

BS: Writing – original draft, Writing – review & editing. LV: Writing – original draft, Writing – review & editing. CD: Writing – original draft, Writing – review & editing. MP: Writing – original draft, Writing – review & editing. HB: Writing – original draft, Writing – review & editing. TT: Writing – original draft, Writing – review & editing. FA: Writing – original draft, Writing – review & editing. JL: Writing – original draft, Writing – review & editing. GW: Writing – original draft, Writing – review & editing. MA: Writing – original draft, Writing – review & editing. BS-E: Writing – original draft, Writing – review & editing. PW: Writing – original draft, Writing – review & editing.
